# Lightweight Cipher for H.264 Videos in the Internet of Multimedia Things with Encryption Space Ratio Diagnostics

**DOI:** 10.3390/s19051228

**Published:** 2019-03-11

**Authors:** Amna Shifa, Mamoona Naveed Asghar, Salma Noor, Neelam Gohar, Martin Fleury

**Affiliations:** 1 Department of Computer Science & IT, The Islamia University of Bahawalpur, Bahawalpur 63100, Pakistan; amna.shifa@gmail.com; 2 Software Research Institute, Athlone Institute of Technology, N37 HD68 Westmeath, Ireland; 3 Department of Computer Science, Shaheed Benazir Bhutto Women University, Peshawar 25000, Pakistan; dr.salmanoor@sbbwu.edu.pk (S.N.); neelam.gohar@sbbwu.edu.pk (N.G.); 4 School of Computer Science and Electronic Engineering, University of Essex, Colchester CO4 3SQ, Essex, UK; fleury.martin55@gmail.com

**Keywords:** encryption space ratio, entropy coding, H.264/AVC, Internet of Multimedia Things, lightweight cipher, selective encryption

## Abstract

Within an Internet of Multimedia Things, the risk of disclosing streamed video content, such as that arising from video surveillance, is of heightened concern. This leads to the encryption of that content. To reduce the overhead and the lack of flexibility arising from full encryption of the content, a good number of selective-encryption algorithms have been proposed in the last decade. Some of them have limitations, in terms of: significant delay due to computational cost, or excess memory utilization, or, despite being energy efficient, not providing a satisfactory level of confidentiality, due to their simplicity. To address such limitations, this paper presents a lightweight selective encryption scheme, in which encoder syntax elements are encrypted with the innovative EXPer (extended permutation with exclusive OR). The selected syntax elements are taken from the final stage of video encoding that is during the entropy coding stage. As a diagnostic tool, the Encryption Space Ratio measures encoding complexity of the video relative to the level of encryption so as to judge the success of the encryption process, according to entropy coder. A detailed comparative analysis of EXPer with other state-of-the-art encryption algorithms confirms that EXPer provides significant confidentiality with a small computational cost and a negligible encryption bitrate overhead. Thus, the results demonstrate that the proposed security scheme is a suitable choice for constrained devices in an Internet of Multimedia Things environment.

## 1. Introduction

An Internet of Things (IoT) is a networked architecture [1], of which the Internet of Multimedia Things (IoMT) [2] is an emerging sub-set, integrating many devices and sensors at the Internet edge. In IoMT applications, video-surveillance devices might be deployed in various scenarios, such as within public transport management systems (managing buses, airplanes or road traffic), health management services (for patient or child monitoring), personal asset protection (within homes or construction sites) and many more [3]. The aim is to make these devices intelligent by allowing them to interact with each other, that is, they become smart objects. Storage and later analysis of data [4] can be on remote cloud data centers. However, even more so than within the traditional Internet, the IoT architecture [5] has inherent security weaknesses of which this paper focuses on confidentiality. Furthermore, the adoption of multimedia rich content, within videos or images, has increased considerably in IoT environments, with the result that the Motion Pictures Experts Group (MPEG) is now standardizing audio–visual and other media data formats [6] as part of an IoMT. The devices in an IoMT, usually rely on Raspberry Pi (RasPi) and complementary metal-oxide semiconductor (CMOS) devices with limited computing and communication capabilities, and, hence, are not powerful enough for complex computations. The adaptation of these limited resource devices in an IoMT, particularly, in surveillance and monitoring systems are constantly increasing. Therefore, these require adequate security measures to keep the information secure. However, in an IoMT, devices have limited resources in terms of processor power and memory and will often be battery powered, there is a requirement for simplified and computationally less complex ciphers. There is also a need to reduce the latency of communication by reducing the overhead arising from full encryption of all the contents. Because full encryption requires decryption at each intermediate point [7], for example if transcoding, splicing of content, adding logos or watermarks needs to take place, not only is there an added computational burden but there is a risk of key disclosure at unsupervised intermediate devices. Thus, full encryption is also an inflexible form of encryption. To cope with these challenges, the technology of lightweight cryptography has been utilized to provide an efficient solution for securing information. Thus, this paper proposes a new lightweight stream cipher which is designed to be implemented easily on surveillance cameras operated with RasPi and the CMOS sensor platform.

This paper assumes that: confidentiality for video sensor networks [8] is integrated into the IoMT and that application-layer encryption is used. The alternative is to rely on any underlying end-to-end security protocol such as transport layer security (TLS) (RFC 6176), itself recently breached, with the overhead of full encryption and an associated public key infrastructure (PKI) network of servers. Consequently, a lightweight encryption cipher, extended permutation with exclusive OR (EXPer), is proposed, which combines statistically random output with efficient encryption, especially if only selected video syntax elements are encrypted. Those video syntax elements, encrypted as part of a selective encryption (SE) scheme, also preserve decoder format compliance, in the sense that the encrypted video can still be processed even if the selected syntax elements are not decrypted [9]. Notice also that if the authors’ contribution in [10] is applied, keys may alternatively be embedded within the encrypted video itself through the joint application of steganography and cryptography, though that aspect is outside the scope of the current paper.

To judge the protection afforded by EXPer, the encryption space ratio (ESR) is calculated as the ratio of the encrypted bits to all the bits of a compressed video bitstream. The ESR has previously been used as one way to judge the effectiveness of SE. In a variable bitrate (VBR) stream, the bitrate is dependent on the various configuration factors but if these are held constant across a number of test videos then both the spatial and temporal coding complexity change according to the content. For example, the presence of high spatial frequencies or textures within objects increases the coding complexity and likewise rapidly moving objects across video frames increases the temporal complexity. The quantization parameter (QP) also affects the bits allocated, with a low QP implying a higher compressed bitrate. The SE bitrate depends on the syntax elements that are selected for encryption. These syntax element bits may be fully encrypted, typically by a block cipher such as the advanced encryption standard (AES) [11] operating in a streaming mode such as cipher feedback (CFB), but, herein, by EXPer for reduced latency. As an example of using the ESR to judge the effectiveness of SE, in [12], nine test videos each encoded with QPs at 16, 24, and 28 (high efficiency video coding (HEVC)) with an ESR ranging from 12.42% to 20.11% and an average of 16.54%, which was judged to be sufficient. In [13] also, the ESR was calculated for a QP of 18 and found to vary from 16.96% to 20.08% depending on the content of six benchmark videos, when using a SE scheme for HEVC. In [13], which provides an analysis of different influences on the ESR, such as the impact of background or HEVC codec profile, the ESR was examined for its diagnostic value. However, the following research questions arise from previous SE-based studies:

**Q1.**  Are those encryption schemes computationally efficient enough (in terms of execution/encoding time) to employ in an IoMT communication environment?
**Q2.**  Is the analyzed ESR effective enough to apply SE to visually secure the videos encoded with one or other of the two common entropy coders in common codec use, that is, context adaptive variable length coding (CAVLC) and context adaptive binary arithmetic coding (CABAC) (see Section 4.1)?

The focus of this paper is to answer those questions by experimentation. In the case of full (sometimes known as) naïve encryption, the complete video is encrypted. Therefore, the encryption overhead and the space ratio are at a maximum, which causes a bitrate overhead too. Herein, such weaknesses are addressed by proposing a complete lightweight security scheme for IoMT applications on a standardized H.264/advanced video coding (AVC) encoder for constrained surveillance devices. Notice that HEVC is currently too resource intensive to be used in an IoMT environment, see [14]. In the proposed scheme, SE is applied through the proposed encryption algorithm EXPer, after identification by means of ESR of an effective level of visual protection. In other words is there sufficient visual distortion within the video frames to preserve the confidentiality of the content. The contributions of the paper are further summarized in Section 1.1. 

### 1.1. Context

SE already has a potential role in consumer electronics applications [15] and also can support interoperability [16], when multiple encryptions of the same video stream are transported. Alternatively, region-of-interest (ROI) encryption of some parts of a video frame such as the face or people within a frame [17,18], may reduce the encryption overhead. However, ROI encryption is application specific, while SE potentially offers a more general solution. Compared to full (or naïve) encryption, both SE and ROI encryption can reduce computational and bitrate overhead [19]. SE may be carried out on the most significant information (as regards distortion) at a choice of different stages of the codec, such as on the original pixels, the transform coefficients, the quantization indexes, the bit-planes, the entropy coder, or the final output bitstream [20]. However, some forms of encryption alter the video statistics, resulting in encryption bitrate overhead and lack of format compliance at the decoder. Applying encryption at the entropy coding stage minimizes those problems [17,18], which is why encryption at that stage of compression is chosen for this paper.

Entropy coders are a feature of standardized hybrid video encoders [21]. H.264/AVC [22] and its scalable video coding (SVC) extension [23,24,25] employ the same entropy coding modes: variable length coding (VLC) or binary arithmetic coding (BAC). Both of these modes operate in a context-adaptive (CA) manner, leading to the names CAVLC [26] and CABAC [27] entropy coders. Within H.264/AVC either CABAC or CAVLC entropy coders can be selected, as the two coders trade-off computational complexity against compression efficiency. The HEVC [28] CABAC coder is a slightly modified version of the H.264/AVC CABAC encoder and, thus, entropy-integrated SE can be configured [13] to work with either codec. However, using HEVC for an IoT is questionable owing to its high computational complexity, except possibly when encoding takes place at a cluster head with maximum energy [29]. 

The H.264/AVC codec is selected for implementation of lightweight encryption because, in both the CCTV industry and for smart monitoring in an IoT, surveillance devices (cameras) mostly operate on microprocessors, especially the RasPi, which only supports video compression in the H.264/AVC format [30]. In an IoT, the RasPi is an economical and privileged platform because it offers a complete Linux server on a tiny platform. To the best of the authors’ knowledge, SE utilized with ESR diagnostics is a novel contribution. In general, the contributions of this paper are:

1. Design of a joint crypto-compression scheme acting upon selected video syntax elements output by the entropy engine of an H.264/AVC encoder. SE is applied, keeping in mind the requirements of IoMT devices.
2. Ensuring that those selected syntax elements do not, once encrypted, have the potential to crash a decoder at the receiver. In other words, ensuring that they are format compatible with the H.264/AVC standard, even when encrypted.
3. Application of ESR to the output from two entropy coders, CAVLC and CABAC. ESR is used as a diagnostic tool to obtain efficient SE. ESR was applied to ten test or benchmark video sequences. (The same procedure has been applied to video clips captured by constrained RasPi cameras.)
4. Finding that the ESR estimate for CABAC is less than CAVLC, which implies that the CABAC entropy coder is suitable for IoMT cameras.
5. Introducing an innovative, single-round, lightweight cipher EXPer (with five sequential steps of block-level eXclusive OR (XOR) cipher and bit-level permutation).
6. Performing a series of experiments with EXPer upon the output of an H.264/AVC encoder, comparing that cipher to simple XOR and the industry-standard advanced encryption standard (AES) [11]. Therefore, three different ciphers are extensively tested (from different aspects) on multiple videos with varying color and motion characteristics. The perceptual strength of the ciphers is compared through different video quality metrics and their computational efficiency is evaluated in terms of execution time on tested videos.
7. Performing cryptanalysis that EXPer is secure against a variety of attacks, including key guessing, perceptual, known-plaintext attacks as well as statistical and inference attacks.

The remainder of this paper is organized as follows. In Section 2, prior efficient lightweight schemes proposed by researchers of IoT are discussed. Section 3 presents the proposed lightweight cipher scheme EXPer, the adopted SE methodology, and diagnostics through ESR. Section 4 describes the promising results over tested videos. Section 5 is a comparative analysis of EXPer with other ciphers (see point 6 of the contributions). Finally, Section 6 rounds off by considering the implications for those planning IoMT video applications with a concern for confidential video content.

## 2. Related Studies 

**Figure 1:**
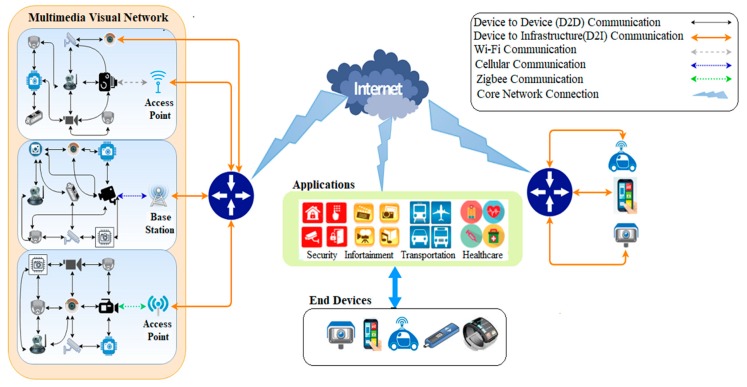
End-to-end communication over multimedia sensor networks in an Internet of Things (IoT).

The heterogeneity of the communication technologies across an IoMT, deriving from the assortment of devices, sensors, and protocols, is a cause of security concern. Messages that are transmitted from smart objects will usually be stored and forwarded through several nodes (such as through video sensors, video relay, transcoders, or other intermediate devices) to reach their endpoint [7]. An endpoint might be a message sink or base station (BS), which passes data to the next layer of the architecture, as occurs in traditional sensor networks when a sink communicates over a satellite link. Figure 1 shows end-to-end communication of multimedia sensor networks within an IoT. Again, there is a need to preserve confidentiality over that link but there is also a need to optimize energy consumption and reduce latency.

To ensure confidential communication of information in an IoT, many schemes have been proposed, schemes which employ existing state-of-art encryption algorithms [31,32,33,34,35,36,37]. Table 1 is a summary of some proposed encryption schemes for IoT with standard cipher algorithms.

In addition encryption, researchers have also proposed authentication schemes for IoT with existing standardized algorithms. Lee et al. [38] proposed a lightweight authentication protocol for RFID systems. In that protocol, privacy protection and anti-counterfeiting was achieved by an encryption algorithm based on XOR manipulation. Mahalle, et al. [39] proposed identity authentication and capability-based access control (IACAC). That scheme provided both authentication and access control for an IoT. However, the scheme results in extra overhead, due to its key-management procedures. The authors of [40] employed encryption and hash algorithms in their proposed solution to achieve confidentiality and message integrity in an IoT. All the same, their solution fails to deal with large amounts of multimedia data because of the proposed encryption algorithm’s ability to encrypt only 64 bits per block; hence, it suffers from slow operation. 

Although, the encryption schemes given in Table 1 provide higher confidentiality, the authors did not consider multimedia content when evaluating the performance of their schemes. Moreover, traditional encryption algorithms, such as AES and triple data encryption standard (DES) encryption, as used in the proposed schemes, are inefficient for an IoMT because of their computationally intensive nature. Hence, those schemes appear to be unsuited to the requirements of real-time IoMT applications, due to their relatively high bitrate overhead, computational overhead, and bandwidth utilization. Consequently, lightweight encryption algorithms are required to alleviate those overheads for low-cost, low-power devices. Recently, there has been much interest shown by researchers and standardization bodies in designing lightweight algorithms for secure end-to-end communication in an IoT. All cryptographic algorithms are based on three principles 1) substitution 2) XOR and 3) permutation. Thus, the newly proposed algorithms by other researchers are also based on these principles [41,42,43,44,45,46,47,48]. Recently, the authors of [48] proposed a one-round cipher (implemented on static images) for IoMT in which the substitution and permutation principles were selected for the encryption. However, substitution is considered resource expensive and should be used with caution over videos, especially for the resource-limited devices of an IoMT. Therefore, to avoid the computational overhead common to substitution, shuffling or permutation is employed in lightweight image encryption algorithms [41,43]. In this current study, a lightweight cipher is designed that additionally employs the XOR principle. It consists of a single round of five sequential steps, (three XOR steps and two permutations). Table 2 is an overview of some recently proposed lightweight encryption algorithms in comparison with our proposed encryption algorithm for IoMT communication.

Likewise, to avoid extra computational overhead and bitrate control, chaos theory is also utilized to implement the encryption process for IoMT systems [49,50]. Chaos theory has proved attractive because of its simplicity and statistical qualities leading to randomized output. Generally, chaotic algorithms are based on a chaotic map and s-box substitution, with multiple rounds to create randomization. However, in the substitution process, multiple rounds to create the desired random output increase the execution time. In fact, in [51] doubt has been cast upon the computational gain from employing chaotic encryption, compared to traditional block-based encryption, such as through AES. Indeed, statistical tests often used to verify the confidentiality of chaotic encryption fail to highlight known insecure encryption algorithms, casting doubt on the claimed security properties. In general, researchers have proposed ciphers for general-purpose applications and do not consider the specific ESR required to provide effective visual protection especially in images and videos.

## 3. Materials and Methods

This section explains the approach of the proposed security scheme for devices in IoMT environment. The security scheme is comprised of four components:

1. Selection of syntax elements for two entropy engines, such as, CAVLC and CABAC.
2. EXPer, an innovative lightweight cipher based on a combination of XOR and bit-level permutation rounds, with three different 128-bit keys.
3. ESR is calculated according to the selected syntax elements of CAVLC and CABAC, as a way of diagnosing the effective visual protection (Section 4).
4. SE is applied by utilizing EXPer, according to the guidance given by ESR (Section 4).

### 3.1. Syntax Elements Selection of Entropy Coders

There are two forms of entropy engines for efficient compression in H.264/AVC video encoder, CAVLC [21] and CABAC [22]. Both CAVLC and CABAC are a lossless form of coding (after earlier lossy encoding stages of the hybrid codec) in which there is a tight data dependency between elements in the output bitstream. CAVLC employs the concatenation of Unary and Exp-Golomb coding for a number of parameters, such as macroblock (MB) type (i.e., the prediction method—inter or intra), coded block pattern (CBP) (which records which blocks within an MB contain non-zero (NZ) transform coefficient (TC) residuals), delta QP, reference frame index, and motion vector differences (MVDs). Quantized transform block coefficients (residuals) are VLC coded after extraction (normally through zigzag scanning) from a block. CABAC gains in efficiency over CAVLC as syntax elements are first converted to a binary format. This allows binary arithmetic coding to be utilized. Arithmetic coding leads to sub-integer probability estimation (unlike CAVLC) but is computationally expensive.

In this paper, for the convenience of implementation the focus is not on a newly proposed SE encoder technique. Therefore, we have employed our previous SE schemes for example as reported in [52], with one newly identified parameter for enhanced visual protection of videos. For the convenience of new researchers, we give simple names to these selected parameters, such as (1) motion, dealing with the movement of objects in videos including camera zooming and other viewing adjustments (2) texture data for pixels information, and a new parameter (3) difference of quantization parameters (deltaQP). Their details can be found in [26,27].

**Figure 2:**
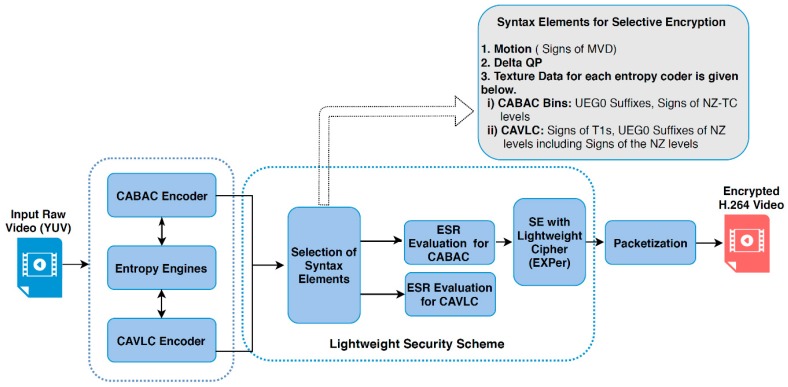
Block diagram of the proposed lightweight security scheme.

It is also worth mentioning here that these mentioned three types of parameters are produced only from residual information, as presented to the final entropy coding stage of a hybrid encoder and are proven to be format compliant in experiments. Textural residuals are taken from both homogeneous and heterogeneous areas within a video. For motion encryption, the arithmetic signs of motion vector difference (MVD) are encrypted while textural syntax elements are different for both CAVLC and CABAC (given in the top grey box of Figure 2), and the absolute values of dQP are selected for encryption. The selected syntax elements of CABAC will be referred as *Bins* in the subsequent sections of this paper. A block diagram of the proposed security scheme is given in Figure 2.

### 3.2. Lightweight Cipher

The proposed lightweight cipher, EXPer, provides both diffusion and confusion primitives, through XORing and permutation. Because the normal substitution process (in mainstream ciphers such as AES) is computationally intensive, so it is not included in the proposed cipher. Moreover, the different forms of XOR are also not computed over videos, because their adoption can be effective for a computation over single image rather than a whole video with large quantity of surveillance frames. Although permutation is applied on bit-level within selected byte to effectively secure the camera captured videos. 

EXPer encryption consists of five steps/stages with a single iteration over those steps. In each step, XOR is performed using a secret key *(k_1)*, and the bit-wise permutation by the shift operation. Permutation is performed with two randomly selected offsets, *v_1, 2,* ranging in value from 1 to 8. Additionally, permutation is performed on the output from the previous stage to provide significant statistical randomness with a reduced computational complexity. The permutation is applied as the bit-level, by re-ordering the bits within each byte. The impact of this permutation is not easily compensated for by an attacker, given that large volumes of video data are involved 

The symmetric secret keys: secret key (*k_1*), sub_key1 *(k_2),* sub_key2 *(k_2)* are dynamically generated at run-time for each input bitstream, by using a pseudo-random function (PRF). To keep the procedure simple, three dynamic keys are generated per video sequence and stored in a registry. Key security can be enhanced by using any standardized key management scheme [53]. Additionally, not one but three 128-bit secret keys have been utilized in the XOR operation. Notice that a key space greater than 2^100^ is considered resilient to key guessing or brute force attacks over keys [54]. Furthermore, selective encryption of selected syntax elements within large volume of videos data has proven to be strong [55] (but see also the updated cryptanalysis of Section 5.5). Moreover, the selected offset values will permute the bits within each byte. We consider the proposed algorithm to be a stream cipher because of the resulting byte-level encryption, in addition to the bit-level XOR operations.

#### 3.2.1. Working of EXPer 

The five steps of the proposed algorithm are discussed in more detail below:

**Step 1:** In the first step, the input bitstream is encrypted by performing an XOR operation with a 128-bit secret key. Let *X* be the selected syntax elements (or bins) of the CABAC entropy coder. Then, *X* is XORed with the secret key, *k_1*. In other words, each 128-bit block of the stream of selected syntax elements, extracted from the original compressed bitstream, is encrypted and then in encrypted form is placed back into the output bitstream:
  *X⊕ k_1 = X’* (1)
  where ⊕ is the XOR operator, *X* contains the syntax elements (or bins) selected from any input bitstream, and *X’* contains the resulting encrypted syntax elements, grouped in blocks of 128-bits.
**Step 2:** In the second step, a permutation is applied to the encrypted output *X’* of (1). Thus, the selected and encrypted syntax elements are re-ordered through the permutation. Specifically, the elements of *X’*, on a byte-by-byte basis, are cyclically permutated by offset value *v_1* using a circular right-shift operator. The shift operation in general is represented in (2), in which the input bytes are transformed, byte-by-byte, into the encrypted and permuted output, as shown in (2) for an offset value one, and in general in (3) by the value of *v_1*
  *(x1, x2, x3…) ↦ (x2, x3, x4…)* (2)
  *X’ >>> v__1_ = X’’* (3)
  where *↦* is a transformation symbol and >>> is a circular shift operator that signifies transforming *X’* into the *X’’* bitstream, through a circular right-shift of the bits of each byte of *X’. v_1* denotes an offset value.
**Step 3:** In the next step, the resulting output of the previous step, which is *X’’*, is again transformed by an XOR operation with the 128-bit sub-key *k_2*, as:
  *X’’⊕ k_2 = X’’’* (4)
  As already mentioned in Section 3.2, *k_2* is derived by means of a PRF.
**Step 4:** In the fourth step, the previously encrypted output *X’’’* is permutated once again with offset value *v_2_,_* again by a circular right-shift operator, applied on a per byte basis:
  *X’’’ >>> v_2 = X’’’’* (5)
**Step 5:** In the final step, the resulting bitstream, *X’’’’*, is XORed with the 128-bit sub_key2 (*k_3*), to produce encrypted bitstream *E_output_*:
  *X’’’’⊕ k_3 = E_output_* (6)
  Subsequently, the bits of encrypted bitstream *E_output_* are re-merged with the compressed video bitstream. In that way, a decoder receives a format compatible bitstream, according to the format of the H.264/AVC standard.

**Figure 3:**
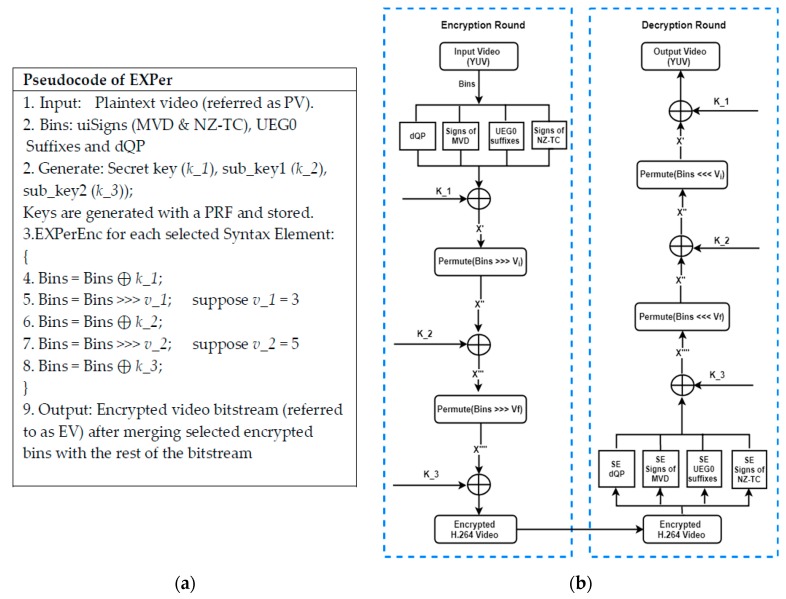
(**a**) Pseudo-code and (**b**) graphical flow of EXPer cipher.

The proposed algorithm is simple and, thus, convenient to implement even on videos directly taken from RaspPi cameras. The pseudo-code and flowchart in Figure 3 demonstrates the simplicity of a software implementation with encryption and decryption rounds.

## 4. Experimental Results and Discussion

In order to evaluate the performance, experiments were performed on ten well-known [56] test videos with varying characteristics, such as slow/fast motion and light/dense colors. The tested videos’ configurations were based upon common intermediate format (CIF) (352 × 288 pixels/frame) at 30 fps, 4:2:0 chroma sampling, IBBP, group of pictures (GoP) frame structure and an intra-refresh period of length 16, with H.264/AVC. The videos are evaluated on different QP values. (The H.264/AVC the range of QPs is from 0 to 51, corresponding to higher compression with lower QPs). All experiments were performed on a 64-bit operating system with 2.30 GHz Core i5-6200U processor and 8 GB RAM. The algorithm was developed using the C/C++ programming language by modifying the JSVM reference software with a single layer [57].

### 4.1. Calculation of ESR for Entropy Coders

Before applying SE with EXPer on test videos, the focus of this paper is to analyze the ESR of two entropy coders over which the SE is applied. ESR is basically the amount of data within each video (calculated in terms of percentages) over which the SE produces acceptable visual protection results (see also Section 1’s introduction to ESR). The ESR percentage is directly proportional to the computational cost of applying SE over videos that is, the more ESR, the more computational cost for SE and vice versa. The ESR for videos calculated on the bases of selected syntax elements (as specified in Section 3.2). The tested videos are listed in Table 3 and configured as described at the beginning of this section.

Taking the ESR percentages for CAVLC first in Table 3, it is apparent that there is a considerable content dependency, probably linked to the spatial complexity of individual video frames and the temporal complexity, due to the level of motion activity within sequences. The ESR percentage of motion elements is much lower than that of the texture ESR, which arises from spatial complexity. However, from the observations of Section 4.1, the ESR value calculated for motion parameter SE elements alone is insufficient in itself to guarantee encryption confidentiality. However, the ESR value for motion and texture SE elements, when those syntax elements are derived from CAVLC can be considerable with a maximum ESR of 29.27% for the Flower test video. It is also the case, that despite the view that SE syntax elements/parameters can be chosen so that in a statistical sense there is little impact on the bitrate overhead, in fact, from experimental evidence, the encryption ratio appears to be considerable.

**Figure 4:**
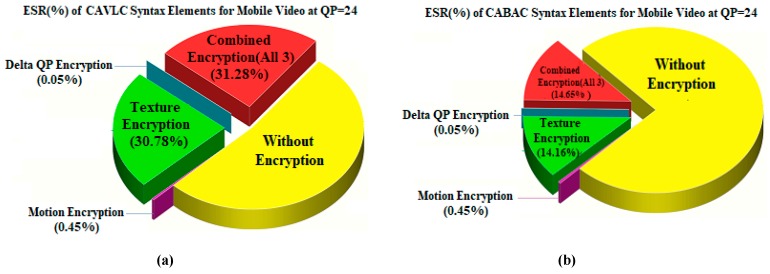
Comparison of calculated ESR over (**a**) CAVLC and (**b**) CABAC syntax elements of mobile video.

Compared with SE of CABAC syntax elements in Table 3, the ESR value is considerably lower for CABAC, with an average (arithmetic mean) of 12% for CAVLC and 7.5% for the CABAC encoded test videos. Additionally, the maximum encryption ratio drops to 13.22% for CABAC and for mobile rather than the flower video. Given that CABAC already has an advantage in terms of compression efficiency (refer back to Section 3), so CAVLC must be adopted with caution for IoT applications. This is unfortunate given the reduced computational overhead arising from CAVLC and its inclusion in H.264/AVC’s baseline-type profiles. The pictorial comparison of ESR calculated for CAVLC and CABAC is illustrated in Figure 4. It is also worth noticing in Table 3 and Figure 4 that for all tested samples, CAVLC produces more texture information than CABAC. This property consequently provides more texture ESR for encryption and, hence, produces more computational overhead than CABAC. For this reason, the EXPer experiments were performed with ESR on CABAC in the next section.

### 4.2. Performance of EXPer

For the evaluation of results with EXPer, the experiments were performed on several test video sequences with the selected parameters such as those based on motion, texture, delta QP, and together with their combinations. The SE is applied with CABAC on all tested videos because CABAC is more compression efficient (refer back to Section 3) and produces less ESR as compared to CAVLC. Another reason for choosing CABAC is that the encryption of the by-pass syntax elements in CABAC does not affect the context models and encrypting at the entropy coder stage does not affect bitstream compliance at the decoder, which is why by-pass CABAC syntax elements are more appropriate for SE in IoMT. Table 4 shows the calculated ESR (ratio) for CABAC syntax elements. 

**Figure 5:**
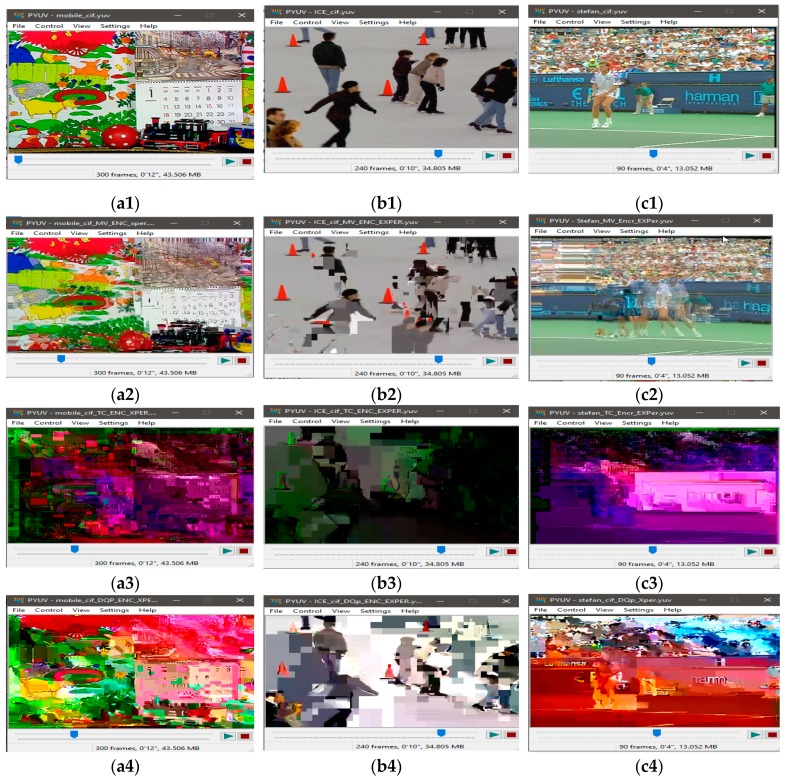

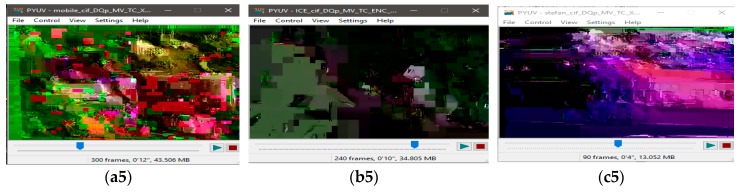
Visual protection of tested videos with EXPer encryption on selected syntax elements. (**a1**) Original mobile video; (**b1**) original ICE video; (**c1**) original STEFAN video; (**a2–c2**) SE on only Motion with EXPer; (**a3–c3**) SE on only texture with EXPer; (**a4–c4**) SE on only delta QP with EXPer; (**a5–c5**) SE on combined (motion, texture and delta QP) parameters with EXPer.

The visual results with the proposed encryption algorithm EXPer on CIF video sequences mobile, ICE and Stefan are presented in Figure 5, which imply that sufficient confidentiality is achieved without generating encryption overhead. The ESR with CABAC for ten videos is depicted in Table 4. The ESR of delta QP is only 0.04% while 0.34% and 6.92% with only motion and texture respectively for the ICE video. The ESR for all parameters for ICE video is 4.25% with the CABAC coder, which is 95.75% less than the data for naïve encryption. The average ESR for test videos with all parameters combined (i.e., motion, texture and delta QP) is 8.69% which is minimal and can be adopted by IoT devices. 

Furthermore, as previously mentioned it is also worth noticing that the ESR with delta QP is comparatively lower than the ESR of only motion or only texture parameters for all tested videos. Thus, the ESR of delta QP combined with motion (0.50% for mobile video and 0.14% for ICE video) or delta QP combined with texture (12.80% for mobile video and 4.15% for ICE video) is also less as compared to the encryption ratio with combined motion and texture (13.22% for mobile video and 4.21% for ICE video). The important point to note here is that the SE on the absolute values of dQP is not possible with complex cipher algorithms, because the number of rounds in complex ciphers makes these values out of range which destroys the format compliance and compression efficiency of the bitstream, and consequently crashes the decoder [52].

However, in this paper, visual results in Figure 5a4,b4,c4 show the effectiveness of EXPer algorithm, as the SE on dQP syntax elements is implemented in a way that their absolute values do not go out of range and, as a result, the compression efficiency and format compliance of videos are both maintained. This format compliance cannot be achieved through the AES algorithm. Overall, the results in Figure 5 indicate that EXPer encryption provides sufficient visual protection with a lower computational and encryption cost than conventional AES encryption.

### 4.3. Computational Cost Analysis

To analyze the performance of the EXPer, the absolute encryption time in seconds was measured for CIF videos. Table 5 shows that the time is negligible compared to the compression time. Thus, the results show that EXPer encrypts the videos with a low computational cost, which is on average 3.1% of the H.264/AVC compression time (when encoded with CABAC) without encryption. Notice that the absolute encryption time is taken separately to the Encoding time (Table 5, column 5)

C*omputational Cost = Encoding Time + Absolute Encryption Time* (7)

### 4.4. Security Analysis of EXPer

The results obtained from various experiments in this Section validate the robustness of EXPer.

#### 4.4.1. Perceptual Security

**Figure 6:**
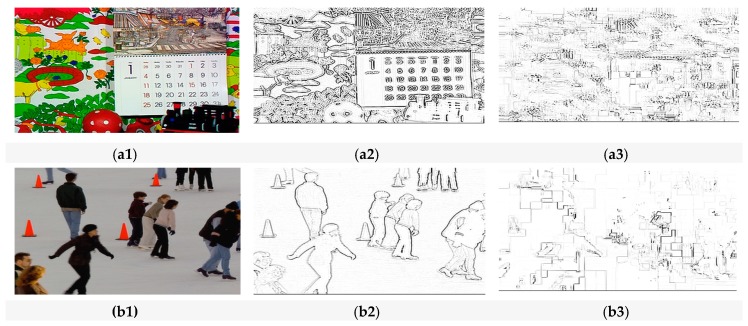
The comparative visual impact of EXPer on ICE and mobile video sequences after Laplacian edge detector. (**a1**) Plaintext mobile video frame; (**a2**) detected edges of plaintext mobile video frame; (**a3**) detected edges of encrypted mobile video frame; (**b1**) plaintext ICE video frame; (**b2**) detected edges of plaintext ICE video frame; (**b3**) detected edges of encrypted ICE video frame.

Perceptual quality is considered an important check on the strength of encryption algorithms. An encryption algorithm is considered robust if it succeeds in distorting a video sequence in such a way that an observer visually fails to detect any useful information from the encrypted bitstream. Clearly, the term ‘useful’ is dependent on the purpose that the video is to be put to, which, herein, is assumed to be IoMT purposes. The visual results of Figure 5 already show that the video sequences encrypted with EXPer produce distorted results compared to the original video sequence. Furthermore, to evaluate the structural distortion of the proposed algorithm, 3 × 3 Laplacian edge detection [58] was performed. The detected edges of the plaintext video and encrypted video frames are illustrated in Figure 6. The comparative results in Figure 6a2,b2 with those of Figure 6a3,b3 show that the SE with EXPer distorts the video in a way that the attacker cannot easily acquire similarity information from edges of the encrypted video. 

#### 4.4.2. Peak signal to noise ratio (PSNR)

PSNR [59] measures the maximum possible absolute differences between the original bitstream and the encrypted bitstream in decibels and is calculated as:

(8) $$\mathrm{PSNR}=10.\log_{10}\frac{{\mathrm{MaxErr}}^{2}}{\sum_{i=1,j=1}^{m,n}\left(X_{i.y}-Y_{i,j}\right)2}$$

where *m* and *n* are the width and height of the video frame under consideration, while *X* and *Y* represent the pixel’s intensity values of the two frames being compared. For a video sequence, the PSNRs of the frames are averaged across the sequence. A lower value of PSNR indicates less similarity between an original video sequence and the video sequence reconstructed from a compressed and encrypted bitstream. 

Table 6 (columns 2, 3) demonstrates the average (arithmetic mean) PSNR of test video sequences after SE (with EXPer) and without encryption (video only compressed). The results show that the average PSNR value is much lower. Hence, the proposed SE with EXPer encryption produced the highly distorted video. Thus, EXPer can be considered for video protection in IoMT.

#### 4.4.3. Pixel-correlation analysis 

Another statistical method to compute the similarity between the original and encrypted pixels of the video frame is cross-correlation. The cross-correlation coefficient, r, is calculated as:

(9) $$r=\frac{\sum_{m}\sum_{n}\left(X_{\mathrm{mn}}-\bar{X}\right)(Y_{\mathrm{mn}}-\bar{Y})}{\sqrt{(\sum_{m}\sum_{n}{\left(X_{\mathrm{mn}}-\bar{X}\right)}^{2})\left(\sum_{m}\sum_{n}{\left(Y_{\mathrm{mn}}-\bar{Y}\right)}^{2}\right)}}$$

where $\bar{X}$, $\bar{Y}$ are the mean intensity values of pixels the original and distorted video frames. The values of the *r* ranges from 1 to −1. When two frames are the same, the correlation index is at a maximum, which is 1. Therefore, a lower value of correlation coefficient indicates higher distortion as a result of encryption. Table 6 (columns 4 and 5) presents calculated cross correlations between encrypted and compressed video frames. The average value of the pixel correlations among the plaintext and encrypted video frames is near to zero, confirming that video sequences encrypted with EXPer are considerably distorted in a statistical sense, thus providing good confidentiality.

**Figure 7:**
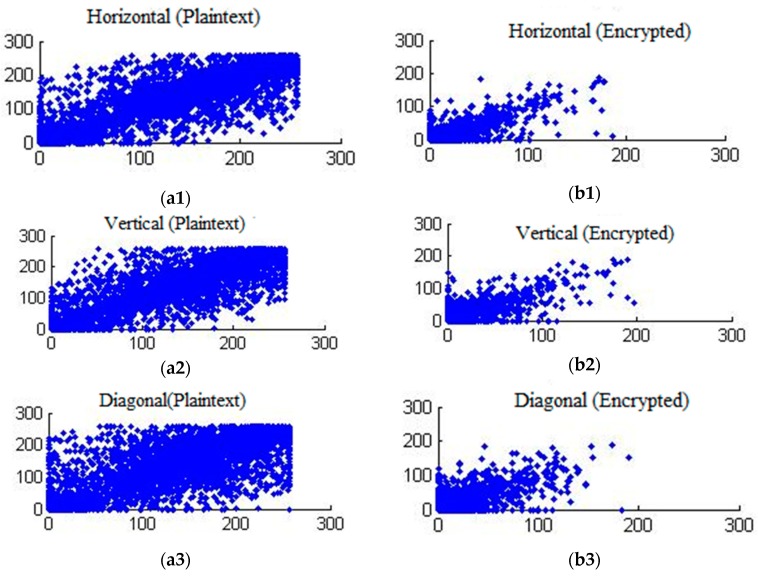
Comparative Pixel correlation of mobile video sequence for plaintext in (**a1**) horizontal direction, (**a2**) vertical direction (**a3**) diagonal direction and when encrypted with EXPer in (**b1**) horizontal direction, (**b2**) vertical direction (**b3**) diagonal direction.

The correlation between adjacent pixels within video frames in the different directions (horizontal, vertical and diagonal) for plaintext and encrypted mobile video are shown in Figure 7. The correlation test is performed by taking randomly *N* = 6000 pairs of adjacent pixels from the original and selectively encrypted test video frames.

#### 4.4.4. Structural Similarity Index (SSIM)

**Figure 8:**
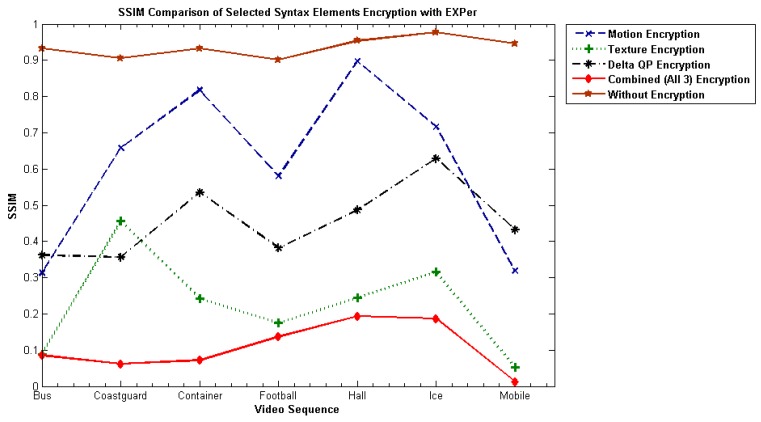
Average structural similarity index (SSIM) values for EXPer on different combinations of motion, texture and deltaQP parameters on test videos.

The SSIM index [60] is a metric which gauges the structural similarity between original and reconstructed video frames, having a range normally from 0 to1. Values of SSIM nearer to 0 means less structural similarity between the plaintext and the reconstructed encrypted bitstream, which means greater distortion has occurred. Values nearer to 1 means more structural similarity. The SSIM values on videos by applying SE with EXPer are reported in Figure 8. The SSIM plots make clear that videos are drastically changed when EXPer is applied on selected combined parameters and that it would be extremely difficult to extrapolate the encrypted parts. 

## 5. Comparison of EXPer with State-of-the-Art Ciphers

To confirm the value of EXPer, a comparison with the most commonly used encryption algorithms XOR and AES was performed. XOR can be considered the most suitable encryption algorithm for IoT applications due to its simplicity and lower computational complexity. However, baseline XOR provides limited confidentiality for images, due to potentially high cross-correlations, and, therefore, AES can be utilized to provide greater confidentiality. Though AES is robust against known-plaintext, brute force, and statistical attacks, it incurs a higher encoding and decoding overhead, which is expensive for resource-constrained IoMT devices. 

**Figure 9:**
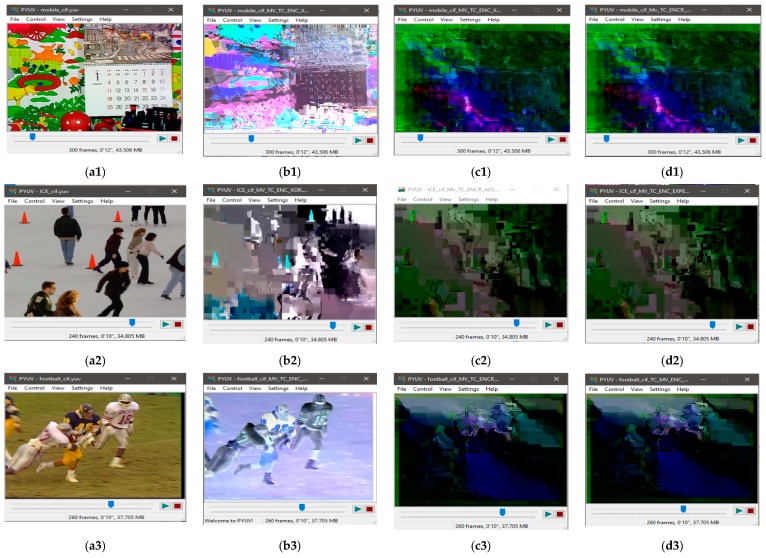
Comparative visual effects of selective encryption (on I, P and B frames) on combined motion + texture syntax elements with XOR, advanced encryption standard (AES)-cipher feedback (CFB) and EXPer on test videos at QP = 36 encoded with CABAC. (**a1**) Original raw mobile video; (**b1**) encryption with XOR; (**c1**) encryption with AES-CFB; (**d1**) encryption with EXPer; (**a2**) original raw ICE video; (**b2**) encryption with XOR; (**c2**) encryption with AES-CFB; (**d2**) encryption with EXPer; (**a3**) original raw football video; (**b3**) encryption with XOR; (**c3**) encryption with AES-CFB; (**d3**) encryption with EXPer.

Results were taken with both state-of-the-art ciphers to compare their performance with EXPer. Comparative visual results with XOR, AES-CFB [11], and EXPer with CABAC coding are presented in Figure 9. Figure 9b1–b3,c1–c3 and d1–d3 depict SE of the videos with three ciphers. The comparative results in Figure 9c vs. d imply that EXPer provides the same level of visual protection and robustness as AES. It is worth mentioning here that dQP encryption is not applied in these comparative results, as AES rounds make the dQP encrypted video non-format compliant.

### 5.1. Comparative Visual Quality Analysis 

For video quality analysis of these three ciphers, PSNR and SSIM results were also taken. Table 7 is a PSNR comparison between XOR, AES-CFB, and EXPer with combined motion and texture parameter encryption on different QPs. The luminance (Y)-PSNR of the mobile video sequence is 7.18 dB, 6.07 dB, and 6.27 dB after encryption with XOR, AES and EXPer respectively. While a noticeable point here is that EXPer is able to encrypt additional syntax element in the implemented SE (Section 4), so the Y-PSNR of EXPer is 6.00 dB (Table 6 (row 4, column 3)), less than AES-CFB, which is 6.07 dB for the mobile video in Table 7. The comparative PSNR results confirm that the proposed algorithm produces PSNR values almost equivalent to AES. 

**Figure 10:**
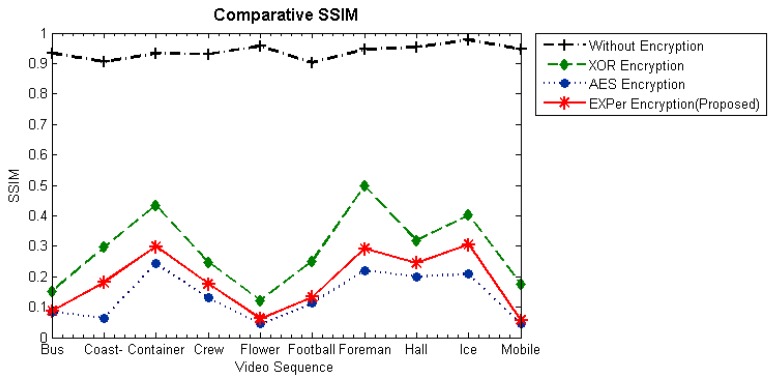
Comparison between SSIM values for XOR, AES-CFB and EXPer for selective encryption with combined motion and texture parameters at QP = 36 on 10 tested video sequences.

The SSIM of the encrypted video with combined motion and texture parameters is illustrated in Figure 9. The comparative results show that video sequences encrypted with EXPer and AES-CFB have smaller SSIM values than encryption with XOR. Lower SSIM values indicate more content protection. Furthermore, from the evidence of Figure 10, EXPer provides almost the same level of confidentiality as AES-CFB. Hence, the PSNR and SSIM results imply that the encryption applied with EXPer provides confidentiality similar to that of AES.

### 5.2. Comparative Computational Efficiency 

**Figure 11:**
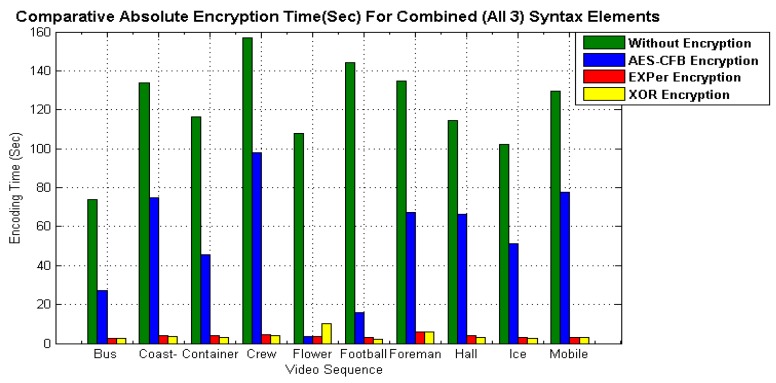
Comparative absolute encoding time of EXPer with AES-CFB and XOR.

In addition to visual content protection, the efficiency of an encryption algorithm for real-time processing is dependent on the execution/encoding time. Therefore, to evaluate the efficiency of EXPer, a comparison with the encoding time of standard algorithms XOR and AES-CFB has been performed. The comparative results of Figure 10 show that the absolute encoding time for only motion parameters encryption and only texture parameters encryption with EXPer is 91.35 s and 87.34 s respectively, which is less than AES-CFB for the ICE video. Likewise, the encoding time for combined motion and texture parameters encryption is 89.41 s, lower than AES-CFB. The graphical results of Figure 11 indicate that the absolute encoding time with EXPer encryption is nearly equivalent to encryption with XOR. Thus, the efficiency of EXPer in terms of execution time is distinctly better than AES-CFB. EXPer provides an almost similar level of protection to that provided by AES-CFB but has a very small computational overhead. 

### 5.3. Comparative Security Analysis 

**Figure 12:**
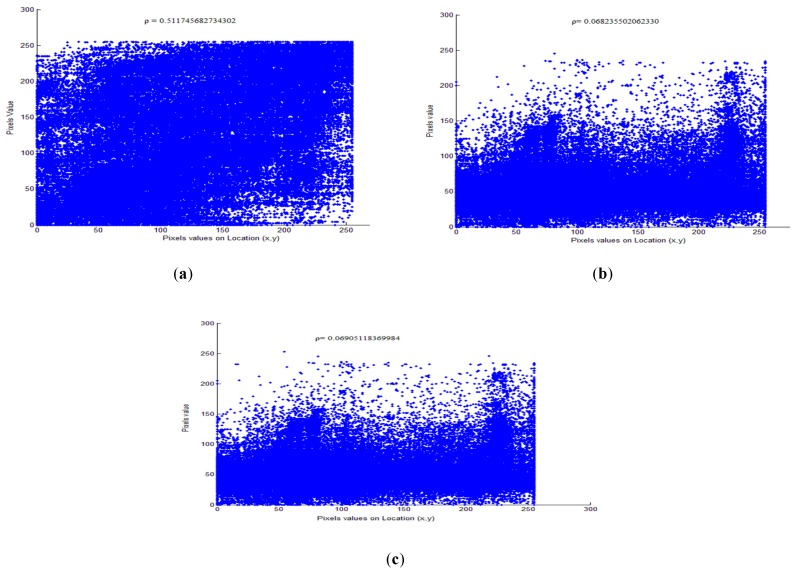
Comparative pixels correlation coefficient of plaintext and encrypted mobile video sequence. (**a**) XOR; (**b**) AES-CFB; (**c**) EXPer.

For the security analysis of EXPer, a comparison with the correlation coefficient of standard algorithms XOR and AES-CFB has been performed. Figure 12 shows comparative correlation coefficients of plaintext mobile video frame and encrypted mobile video frame. The results show that frame encrypted with the EXPer has pixel correlation coefficient value ρ = 0.06905118369984 which is almost equivalent to the pixel correlation value of the frame encrypted with AES-CFB, which is 0.068235502062. This result implies that EXPer has achieved the same level of randomness as AES-CFB and outperform baseline XOR encryption, which has correlation value ρ = 0.511745682734. This demonstrates that the proposed EXPer has a greater potential to resist statistical attacks.

### 5.4. Comparative Entropy Analysis

**Figure 13:**
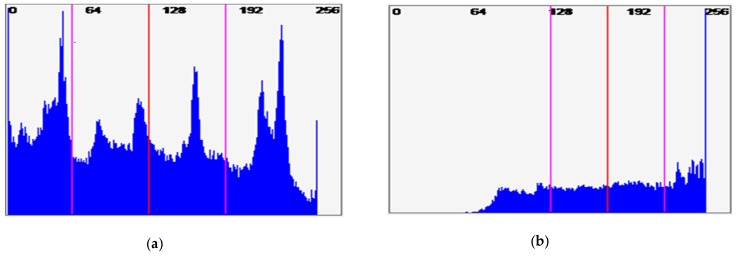

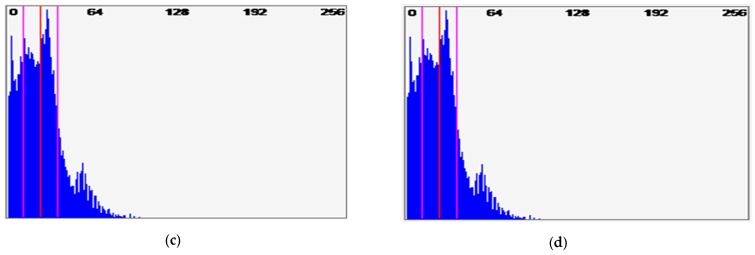
Comparative entropy of plaintext and encrypted mobile video. (**a**) Original frame (Frame# 123); (**b**) encrypted with XOR; (**c**) encrypted with AES; (**d**) encrypted with EXPer (Proposed).

Entropy defines the uncertainty or the chaos level within video frames. It measures the amount of the gray level and the probability corresponding to the total information inside all other pixels and determines which pixels carry most of the information. It is calculated as:

(10) $$H\left(f\right)=-\overset{2N-1}{\underset{i=0}{\sum }}p\left(f\right)\log_{2}\left(f\right)$$

where *f* is the gray-level value and *p(f)* is the probability of *f*. Figure 13 is a comparative entropy histogram of mobile video frames encrypted with XOR, AES and EXPer. This entropy is evident in the spreading of more black and sharp colors (shown in Figure 5; Figure 9) across the video frames, compared to the original histogram values (Figure 13a) prior to applying SE with either one of the three ciphers to the selected syntax elements taken from the mobile video sequence. 

### 5.5. Cryptanalysis of Proposed ESR-Validated Security Scheme

This section contains a cryptanalysis of relevant attacks upon the SE method of this paper. A prior cryptanalysis of the SE method also appeared in the leading journal paper [55], reviewed by security specialists but aimed at a multimedia audience. Therefore, we now include an additional analysis, including an analysis of further attacks. 

#### 5.5.1. Differential Attack 

**Figure 14:**
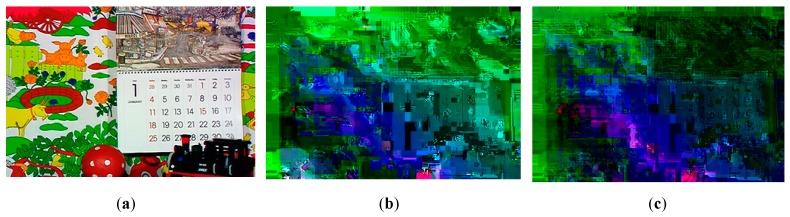
Key sensitivity analysis. (**a**) Original frame; (**b**) decrypted with K1; (**c**) decrypted with K2.

In this type of attack, the attacker tries to guess the keys and sub-keys by investigation of the encrypted bitstream streams. However, the proposed algorithm is sensitive to any changes in the plaintext bitstream or in the secret keys or the offset value. Bit-level chaining dependency exists between the permutation and XOR sequential steps in EXPer, which makes it challenging to apply differential attacks. Hence the proposed algorithm produces a different encrypted output bitstream for the same plaintext input bitstream if any change in the secret key takes place. Thus, whenever an attacker tries to insert random keys through a brute force attack in order to recover the original bitstream, they find more distorted bitstream rather on any clues about the encrypted bitstream and the sub-keys, as demonstrated in Figure 14.

#### 5.5.2. Known-Plaintext and Correlation Attack 

EXPer is implemented on video syntax elements rather than on a single image. All tested videos of file size 43.5 (Table 3) are comprised of 300 frames. Table 3; Table 4 show that the tested mobile video has an overall ESR equal to 13.26%, based on a total number of encrypted 6,057,747 syntax elements and exploitation of all the important video characteristics, such as motion, texture characteristics, and QP variable length values. This total is a large number of syntax elements, distributed across 300 frames. This number of elements and their distribution makes it very difficult for attackers to guess the values of those syntax elements correctly so as to render the encrypted video watchable. The syntax elements are independently encrypted and have no correlation with each other. Motion vectors only deal with motion of the video, while transform coefficients only affect the spatial resolution of the video, while the QP values are for controlling the bitrate. Therefore, MVD, TC sign encryption and dQP have no relation to each other. Consequently, correlation attacks have little hope of success.

#### 5.5.3. Interference Attack 

The entropy analysis performed in Section 5.4 implies that, if the videos are selectively encrypted by EXPer, they are highly distorted and it is difficult for an attacker to infer the presence of an object in any one of the R, G, B and luminance domains. The results, previously shown in Figure 13 for entropy, illustrate that the EXPer has attained confidentiality against inference attacks equivalent to encryption with AES-CFB.

### 5.6. Discussion and Limitations 

A comparison of two recently proposed lightweight ciphers with EXPer appears in Table 2. Encryption is a trade-off between speed of computation and resistance to cryptanalysis. In [48] the authors selected the substitution and permutation principle (refer back to Section 2) for the encryption of static images for an IoT. However, substitution cannot be efficiently computed within the resource-limited devices of an IoMT specifically for videos and, therefore, should be used with caution. Likewise, the authors of [41,43] only incorporated shuffling or the permutation principle in their ciphers. However, those ciphers, based as they are on shuffling alone, are vulnerable to attack [61,62]. Similarly, XOR is considered as weak in security terms. However, it may be suitable in some circumstances within an IoMT, due to its simple implementation and fast execution. Moreover, a single permutation of the key makes it vulnerable to many attacks such as known-plaintext attacks. It is for that reason that the proposed EXPer is employed with XOR and permutations in successive rounds to obtain adequate confidentiality, in the sense that lightweight ciphers must trade-off real-time operation and complete invulnerability. Additionally, a 128-bit secret key has been utilized, which length is considered unbreakable until 2020 is reached (by virtue of the time needed to test every possibility). Table 8 shows the comparative analysis of EXPer with respect to the visual quality metrics with other two schemes.

## 6. Conclusions

Various classes of IoMT devices are utilized for multiple services such as stored video streaming (YouTube, online lectures), live video streaming in the cases of video conferencing, online gaming, and other real-time applications, with the most confidential being interactive video streaming in the form of surveillance applications. It is crucial to modern IoMT nodes to provide data confidentiality in the form of data encryption. The most reliable cipher is AES with 128/192/256 bit keys. However, AES is still not an optimal choice for low-powered surveillance devices with simple hardware. In this paper, by keeping in view current security needs, we propose an ESR-validated security scheme for IoMT devices. Within a security scheme, the contribution of the paper is to examine two alternative entropy coders available for the H.264/AVC codec, such as AVLC and CABAC in detail and determine the ESR when applying SE with the proposed cipher to encrypt the selected syntax elements. As identified herein, using the CABAC entropy coder (see Table 3) can considerably save on the ESR percentage that is the maximum is 14.14%, while the CAVLC is 31.23%, so that the equivalent average ESR is 12% for CAVLC and 7.5% for CABAC across the tested reference videos. The ESR calculated for CABAC is acceptable for IoMT applications as reduced encryption data consumes less computation during encoding. In the proposed security scheme, a novel cipher, EXPer, works on cryptographic basic principles such as permutation and XOR with three different 128-bit keys over selected syntax elements of CABAC encoder. EXPer even performs very well on absolute values of syntax elements that is dQP, without changing the bitrate and crashing the decoder (if the decoder is applied to encrypted video). Comparative analysis with the existing state-of-art ciphers shows that EXPer yields confidentiality almost similar to that of AES-CFB, but the computational cost is similar to the XOR, which makes it a suitable choice for protecting real-time video communication in an IoMT setting. Our detailed security analysis revealed that the proposed EXPer is robust enough against multiple attacks. 

Future work based on taking measurements of ESR in this paper can provide a way to more precisely model the trade-offs between computational complexity, and memory access in terms of energy consumption within a video sensor device. Both entropy coders have a content dependency, which increases the effect of bit errors. This implies that error resiliency or channel coding should be built into transmission over an IoMT network along with proper key management solution in future.

## Figures and Tables

**Table 1:** Summary of recently proposed security schemes for IoT by using existing standardized encryption algorithms.

Proposed Scheme	Year	Platform	Algorithms Used	Strengths	Limitations
El Assad S, Farajallah M [33]	2016	FPGA card or an ASIC/	Diffusion and chaotic map (2D cat map)	Efficient and provides: A high-level of security, resistance to known plaintext and chosen plaintext attacks	Requires a huge memory capacity
Al-Salami et al. [34]	2016	Smart Home	Identity-based encryption (IBE), Stateful Diffie-Hellman (DH) Encryption, Private Key Generator (PKI)	Provides favorable computational and communication efficiency	Overhead of handling private key generator (PKG)
Yao et al. [35]	2015	IoT	Attribute-Based Encryption (ABE), Elliptic Curve Cryptography (ECC), DH, Elliptic Curve Decisional Diffie-Hellman (ECDDHP)	Improved execution efficiency and reduced communication costs	Poor flexibility in revoking attributes and weak scalability
Xin M [36]	2015	IoT	MD5, ECC, and AES algorithms	Improved security and performance	Increases the complexity and execution speed
Prasetyo et al. [37]	2014	FPGA module/constrained devices	Blowfish algorithm	Better security and reduced total encryption time	Larger key length requires more resources and suffers from error propagation

**Table 2:** Comparative overview of existing lightweight ciphers for constrained devices and Extended Permutation with eXclusive OR (EXPer).

Algorithm	Target Multimedia	Structure	Target Devices/Platform	Cipher Type	Key Size (bits)	No. of Rounds	Secure Against These Attacks
Total shuffling matrix (2006) [41]	Image	Permutation and XOR (Shuffling with a combination of two chaotic streams)	Internet	Not specified	84	Multiple iterations	Brute-force attacks
Total shuffling scheme {2011) [43]	Image	Permutation–diffusion	Internet	Block	104	1	Differential attacks Chosen- plaintext & Known-plaintext attacks
Hummingbird2 (2012) [44]	General purpose	Hybrid	Low-end controllers, RFID tags, wireless sensors, smart meters	Hybrid	256	4	Related-key attack Side-channel attacks
TWINE (2013) [45]	General purpose	Type-2 generalized Feistel network (GFN-2)	Micro-controller and high-end CPU	Block	80/128	32	Meet-in-the-Middle attacks
PRIDE(2014) [46]	General purpose	Substitution permutation network	8-bit micro-controller	Block	128	20	Meet-in-the-Middle attacks, Differential attacks
Lightweight chaotic image encryption algorithm (2018) [47]	Image	Chaotic map	32-bit microcontroller and real-time embedded applications	Block	128	Not specified	Not specified
One round encryption algorithm (2018) [48]	Image	Substitution permutation network	Multimedia IoT	Stream	512	1	Key-related, Chosen-plaintext, Known–plaintext attacks
Proposed EXPer (2019)	H.264/AVC videos	Bit-wise permutation and XOR over CABAC encoded syntax elements (Bins)	Multimedia IoMT	Stream	128	1	Perceptual, Key guessing, Known-plaintext, Differential, Statistical & Inference attacks

**Table 3:** Comparative encryption space ratio (ESR) (%) of context adaptive variable length coding (CAVLC) and context adaptive binary arithmetic coding (CABAC) syntax elements for sample videos at quantization parameter (QP) = 24.

Sr. #	Videos (CIF)	File Size (MB)	Encryption Ratio (%)					
			Only Motion Encrypted		Only Texture Encrypted		Combined (Motion and Texture) Encryption	
			CAVLC	CABAC	CAVLC	CABAC	CAVLC	CABAC
1.	Bus	21.7	0.55%	0.56%	23.42%	10.72%	23.97%	11.28%
2.	Coastguard	43.5	0.32%	0.25%	21.52%	4.46%	21.84%	4.71%
3.	Container	43.5	0.07%	0.32%	6.94%	10.10%	7.01%	10.42%
4.	Crew	43.5	0.45%	0.07%	12.57%	3.17%	13.01%	3.24%
5.	Flower	36.3	0.56%	0.45%	28.71%	7.57%	29.27%	8.02%
6.	Football	37.7	0.52%	0.57%	25.79%	13.57%	26.30%	14.14%
7.	Foreman	43.5	0.30%	0.52%	11.61%	11.30%	11.92%	11.82%
8.	Hall	36.3	0.10%	0.30%	11.11%	5.05%	11.21%	5.35%
9.	ICE	34.8	0.34%	0.10%	6.92%	4.11%	7.26%	4.21%
10.	Mobile	43.5	0.45%	0.46%	30.78%	12.76%	31.23%	13.22%

**Table 4:** ESR in terms of percentage over which selective encryption (SE) applied on CABAC bin-strings at QP = 24.

Sr.#	Videos (CIF)	File Size (MB)	ESR (%)						
			Only Motion	Only Texture	Only Delta QP	Both Motion and Delta QP	Both Texture and Delta QP	Both Motion and Texture	Motion, Texture and Delta QP
1.	Bus	21.7	0.56%	10.72%	0.06%	0.62%	10.78%	11.28%	11.34%
2.	Coastguard	43.5	0.25%	4.46%	0.04%	0.29%	4.50%	4.71%	4.75%
3.	Container	43.5	0.32%	10.10%	0.02%	0.34%	10.12%	10.42%	10.44%
4.	Crew	43.5	0.07%	3.17%	0.08%	0.15%	3.25%	3.24%	3.32%
5.	Flower	36.3	0.45%	7.57%	0.05%	0.50%	7.62%	8.02%	8.07%
6.	Football	37.7	0.57%	13.57%	0.10%	0.67%	13.67%	14.14%	14.24%
7.	Foreman	43.5	0.52%	11.30%	0.03%	0.55%	11.33%	11.82%	11.85%
8.	Hall	36.3	0.30%	5.05%	0.03%	0.33%	5.08%	5.35%	5.38%
9.	ICE	34.8	0.10%	4.11%	0.04%	0.14%	4.15%	4.21%	4.25%
10	Mobile	43.5	0.46%	12.76%	0.04%	0.50%	12.80%	13.22%	13.26%

**Table 5:** Absolute encryption time (s) with H.264/AVC CABAC entropy coding on selected parameters (motion, texture, delta QP and their combination) with EXPer.

Sr. #	Video (CIF)	File Size (MB)	Encoding time with H.264/AVC Using CABAC (Compression Without Encryption)	Absolute Encryption Time by Applying SE on CABAC (s)						
				Only Motion	Only Texture	Only Delta QP	Motion and Texture	Motion and Delta QP	Delta QP and Texture	Motion Texture and Delta QP
1.	Bus	21.7	73.697 s	1.896	1.044	1.195	1.249	0.342	1.433	2.431
2.	Coastguard	43.5	133.828 s	1.622	0.177	4.045	3.655	3.831	3.528	4.012
3.	Container	43.5	116.386 s	2.687	2.691	2.656	3.217	3.058	2.666	3.947
4.	Crew	43.5	156.804 s	1.601	1.803	4.151	2.928	3.373	3.079	4.512
5.	Flower	36.3	107.977 s	1.849	2.745	1.059	2.908	0.143	3.561	3.772
6.	Football	37.7	143.975 s	1.154	1.149	2.995	1.866	0.129	0.989	2.958
7.	Foreman	43.5	134.769 s	2.419	2.285	2.793	0.993	5.483	3.603	5.988
8.	Hall	36.3	118.614 s	1.198	3.267	3.554	3.141	3.611	3.25	3.984
9.	ICE	34.8	105.232 s	0.947	2.694	3.54	3.4	2.998	2.625	3.229
10.	Mobile	43.5	129.394 s	1.786	2.773	2.809	0.893	3.683	2.038	3.186
Average absolute encryption time (s):				1.715	2.062	2.879	2.226	2.639	2.677	3.801

**Table 6:** Comparison of average peak signal to noise ratio (PSNR) (dB) and pixel cross-correlation of SE at QP = 36 with EXPer for the videos encoded with CABAC.

Test Videos	Average PSNR (dB)		Average Pixel Cross-Correlation	
	Compressed	Encrypted	Compressed	Encrypted
Football	[Y:31.63,U:37.45,V:39.01]	[Y:10.56,U:18.97,V:19.25]	0.9816	0.0471
ICE	[Y:29.18,U:34.16,V:33.47]	[Y:6.27,U: 14.99,V:11.64]	0.9991	−0.0173
Mobile	[Y:32.86,U:38.06,V:39.47]	[Y:6.00,U: 21.97,V:27.32]	0.9923	0.0140

**Table 7:** Comparison of average PSNR (dB) of SE with XOR, AES-CFB and EXPer at four different QP levels.

Videos	QP	Encoded Without SE			SE with XOR			SE with AES-CFB			SE with EXPer		
		Y	U	V	Y	U	V	Y	U	V	Y	U	V
Football	12	43.9	46.71	47.65	9.72	13.08	20.92	9.52	18.25	21.13	9.53	19.25	22.13
	24	37.03	41.55	42.76	8.67	13.28	20.75	10.04	18.06	19.36	10.16	19.06	20.36
	36	31.63	37.45	39.01	9.94	13.19	20.87	10.36	17.97	18.24	10.56	18.97	19.25
	48	28.72	34.99	37.1	10.63	12.94	21.07	11.31	17.38	16.9	11.51	18.4	17.96
Mobile	12	42.72	45.18	44.96	7.05	13.26	13.95	6.07	12.47	10.46	6.00	13.47	11.46
	24	34.69	38.46	38.03	7.09	13.2	14.04	6.06	13.18	10.79	6.19	14.18	11.79
	36	29.18	34.16	33.47	7.18	13.09	14.16	6.07	13.98	10.62	6.27	14.99	11.64
	48	24.96	31.89	31.01	7.54	14.83	13.97	7.05	15.3	11.83	7.08	16.35	12.84
ICE	12	44.74	48.77	49.56	7.91	18.82	25.85	5.74	20.39	26.43	5.94	21.39	27.43
	24	38.78	43.21	44.02	7.73	18.33	26.64	5.8	20.97	26.32	6	21.97	27.32
	36	32.86	38.06	39.47	7.93	18.74	27	6.01	21.98	23.75	6.07	22.98	24.77
	48	29.09	36.03	37.58	8.25	18.21	27.66	5.9	18.54	19.25	5.98	19.57	20.27

**Table 8:** Comparison of average PSNR, SSIM and entropy with that of other lightweight ciphers.

Sr. No	Entropy	PSNR (dB)	SSIM
Zhang et al. [43]	7.997	Not specified	Not specified
Noura et al. [48]	5.7566	8.5894	0.034
Proposed EXPer	5.1265	7.9325	0.183
